# Ecofriendly Approach for Treatment of Heavy-Metal-Contaminated Water Using Activated Carbon of Kernel Shell of Oil Palm

**DOI:** 10.3390/ma13112627

**Published:** 2020-06-09

**Authors:** Rabia Baby, Mohd Zobir Hussein

**Affiliations:** 1 Material Synthesis and Characterization Laboratory, Institute of Advanced Technology, Universiti Putra Malaysia, Serdang 43400, Selangor, Malaysia; rabia.shaikh@iba-suk.edu.pk; 2 Department of Education, Sukkur IBA University, Sukkur 65200, Pakistan

**Keywords:** activated carbon, water treatment, heavy metal, greener method, palm oil kernel shell

## Abstract

Heavy metal ion contamination in water poses a significant risk to human health as well as to the environment. Millions of tons of agricultural wastes are produced from oil palm plantations which are challenging to manage. In this study, we converted palm kernel shells (PKS) from a palm oil plantation into activated carbon (AC) having a surface area of 1099 m^2^/g using phosphoric acid as an activator. The prepared material was characterized using BET, XRD, Raman, FESEM and FTIR analyses. The AC was applied for the treatment of heavy-metal-contaminated water, and different parameters; the pH, adsorbent dosage, contact time and metal ion concentrations were varied to determine the optimal conditions for the metal ion adsorption. Different kinetic models; the zeroth, first-order and second-order, and Freundlich and Langmuir isotherm models were used to determine the mechanism of metal ion adsorption by the AC. Under the optimized conditions, Cr^6+^ and Pb^2+^ were removed completely, while Zn^2+^ and Cd^2+^ were more than 80% removed. This is a greener approach in which an agricultural waste, PKS is converted into a useful product, activated carbon and subsequently applied for the treatment of heavy metal-contaminated water.

## 1. Introduction 

Life is unheard of without the presence of water on any planet. The industrialization has immensely contributed to environmental pollution, notably causing water contamination [1,2]. According to a WHO 2019 report, about 785 million people are deprived of basic drinking water services, and nearly 2 billion people are drinking contaminated water. The use of contaminated water transmits diseases such as cholera, diarrhea, typhoid, dysentery and polio [3,4]. In addition to drinking water, the contamination of irrigation water also affects human health [5]. Various water contaminants exist, namely viruses, bacteria, dyes, organic molecules and toxic heavy metal ions [4,6]. All contaminants are biodegradable except heavy metal ions, which in addition to being nonbiodegradable, accumulate in the body [7,8,9]. Different industrial activities are responsible for heavy metal contamination in water, such as the mining, smelting, fertilizer and electroplating industries [2,4,6,8,9]. Agricultural products are ultimately consumed by humans and living creatures, resulting in health problems [1,7,10]. The consumption of heavy-metal-contaminated water and foods can cause skin diseases, brain damage, liver damage, anemia, ulcers, hepatitis and cancer, etc. [1,11,12,13]. Several methods exist for the purification of heavy-metal-contaminated water, e.g., evaporation-condensation, reverse osmosis, ion exchange, membrane filtration, coagulation and adsorption process [1,6,14]. Among these methods, adsorption has many advantages such as good efficiency, the stability of the adsorbents, low cost and minimum energy consumption, also it is easier and user-friendly [1,6,14,15]. Adsorption processes involve adsorption–complexation, chemisorption, complexation and physio-sorption processes [1,14,16]. A variety of adsorbent materials are being employed, such as clay materials, bio-sorbents, plants, agricultural biowaste and metal oxides. [1,6,14,16]. Nanotechnology platforms offer a variety of solutions to environmental challenges by controlling the design and synthesis of nanomaterials with unique shapes, sizes and pore sizes [14,17,18,19,20]. A variety of carbon nanomaterials exist, e.g., fullerenes, single-wall carbon nanotubes, multiwall carbon nanotubes, graphene and activated carbon. All these nanomaterials offer advantages but, except for activated carbon, they are expensive and difficult to use on a large scale, [14,21,22,23]. Activated carbon is non-toxic, possesses high surface area and is environmentally friendly [14,19]. Activation yields various advantages e.g., high adsorption capacity due to its pore structure and high surface area. A rich carbon mass could be utilized by processes through carbonization and activation, yielding the activated carbon. The carbon has a porous composition, which increases its adsorption characteristics either by physical or chemical activation. The process involves applying temperatures of 500–1000 °C to a carbon-rich mass in the absence of oxygen (O_2_), producing a carbonaceous mass, i.e., charcoal [24]. 

Agricultural waste is a sustainable and reproducible material which can be utilized to produce carbonaceous mass, so-called activated carbon [6,14]. Palm oil kernel shells (PKS) are a form of agricultural waste from oil palm plantations, millions of tons of which are produced every year [1,18]. In this study, PKS was used as the source of carbon and phosphoric acid (H_3_PO_4_) was utilized for the activation of the carbon-rich mass. The synthesized activated carbon was applied for the removal of heavy metal ions from water by the adsorption process.

## 2. Materials and Methods

### 2.1. Chemicals 

Phosphoric acid (H_3_PO_4_), Standards solution of Cr^6+^, Cd^2+^, Zn^2+^, and Pb^2+^ (Sigma-Aldrich, St. Louis, MO, USA) and deionized water were used in this study.

### 2.2. Pretreatment of and Activation of Sample

The samples of PKS were obtained from a palm oil mill, Dengkil, Malaysia. The PKS was thoroughly washed with deionized water and the sample was dried in an oven at 60 °C. The PKS samples were ground to powder and is then treated with phosphoric acid for activation. The activation of the sample was carried out using the phosphoric acid method. Briefly, about 20 g of the sample was pretreated with different concentrations of phosphoric acid: i.e., 0%, 5%, 10%, 20%, 30% and 40% (V/V) [18]. The sample was shaken at 120 rpm for 24 h, followed by filtration and oven drying at 70 °C [18]. A tubular electric furnace was utilized for the activation/carbonization process with a constant flow of nitrogen at 150 cm^3^/min. Parameters such as temperature, holding time and phosphoric acid concentrations were varied to get the highest surface area of the sample. 

### 2.3. Effect of Temperature, Holding Time and H_3_PO_4_ Concentration on the Carbonization

To determine the optimized temperature, about 5 g activated PKS sample was carbonized in a tubular furnace under N_2_ gas. The PKS activated with 20% phosphoric acid was selected to study the temperature effect on carbonization. Different temperatures, i.e., 500 °C, 600 °C, 700 °C, 800 °C and 900 °C were applied for a 2-hour holding time with a heating rate of 10 °C/min. The resulting samples were dried at 100 °C to eliminate moisture. Different holding times were applied, i.e., 1 h, 2 h, 3 h, 4 h and 5 h. Different concentrations of H_3_PO_4_, i.e., 0%, 5%, 10%, 20%, 30%, and 40% were also used to determine the effect of concentration on the activation. 

### 2.4. Instrumentation

Raman Spectrometer (WiTec, Ulm, Germany), XRD (Shimadzu, Kyoto, Japan), FTIR spectrometer (Perkin-Elmer 100 series, Waltham, MA, USA), Field emission scanning electron microscope (FESEM) JOEL JSM-6400 (Tokyo, Japan) and inductively coupled plasma-Optical Emission Spectrometer (ICP-OES), Optima 2100 DV Perkin Elmer (Waltham, MA, USA) were used for the characterization of the samples. 

### 2.5. Characterization

Functional group analysis was carried out using a Fourier transformed infrared (FTIR) spectrometer, Perkin-Elmer 100 series (Waltham, MA, USA). Surface morphology was determined using a field emission scanning electron microscope (FESEM), JOEL JSM-6400 (Tokyo, Japan). Elemental analysis was done using an inductively coupled plasma-optical emission spectrometer (ICP-OES), Optima 2100 DV Perkin Elmer.

## 3. Results and Discussion

### 3.1. Surface Area and Porosity 

**Figure 1:**
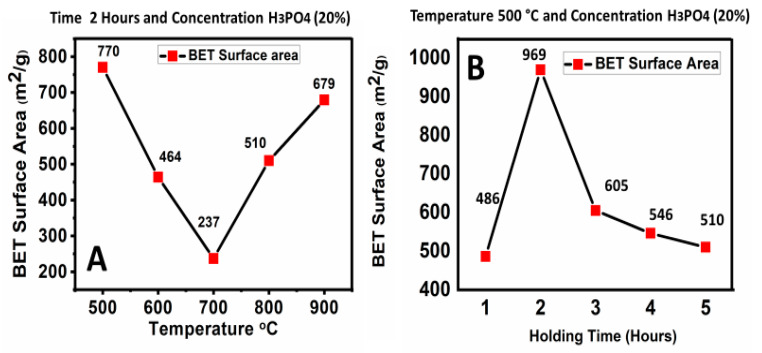

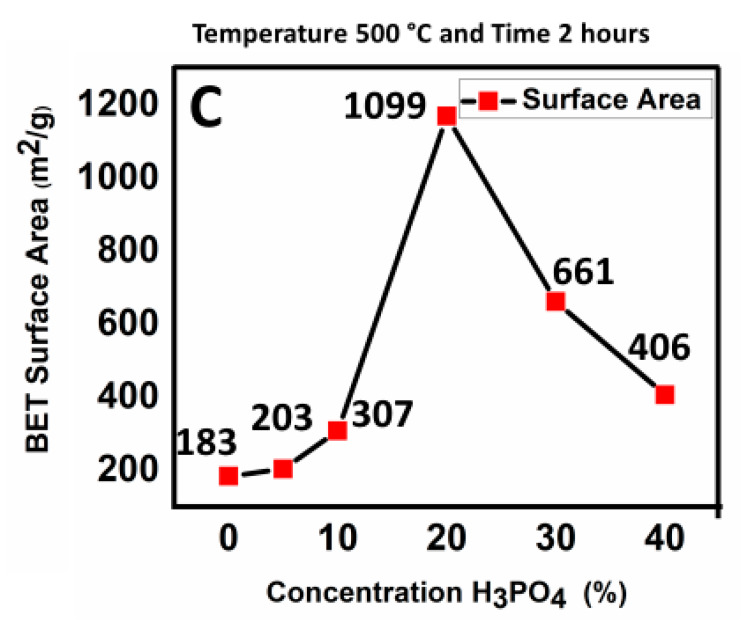
Optimization of temperature, holding time and concentration of phosphoric acid in the synthesis of activated carbon with a flow rate of 1 cm^3^/min of nitrogen gas, showing the effect of temperature (**A**), the effect of holding time (**B**) and the effect of concentration (**C**).

The parameters for the synthesis of activated carbons, i.e., temperature, holding time and concentration of activator phosphoric acid, were optimized in our previous work [18]. Briefly, 500 °C, 600 °C, 700 °C, 800 °C and 900 °C were applied to determine the optimal temperature for the synthesis of activated carbon. The activated carbon formed at 500 °C was found to have the highest surface area, 770 m^2^/g, and this temperature was then used for the optimization of the remaining parameters. Different holding times were applied, i.e., 1 h, 2 h, 3 h, 4 h and 5 h; the best sample with the highest surface area (969 cm^2^/g) was produced at a holding time of 2 h. Then different concentrations of activator, phosphoric acid (H_3_PO_4_) i.e., 0%, 10%, 20%, 30% and 40%, were utilized, keeping the previously optimized temperature and holding time constant. At a 20% concentration of phosphoric acid, the surface area was found to be optimum, i.e., 1099 m^2^/g. Figure 1A–C show the optimization results for temperature, holding time and concentration of phosphoric acid, respectively. This sample of activated carbon was utilized for the adsorption batch experiments.

### 3.2. Adsorption and Desorption Isotherms 

**Figure 2:**
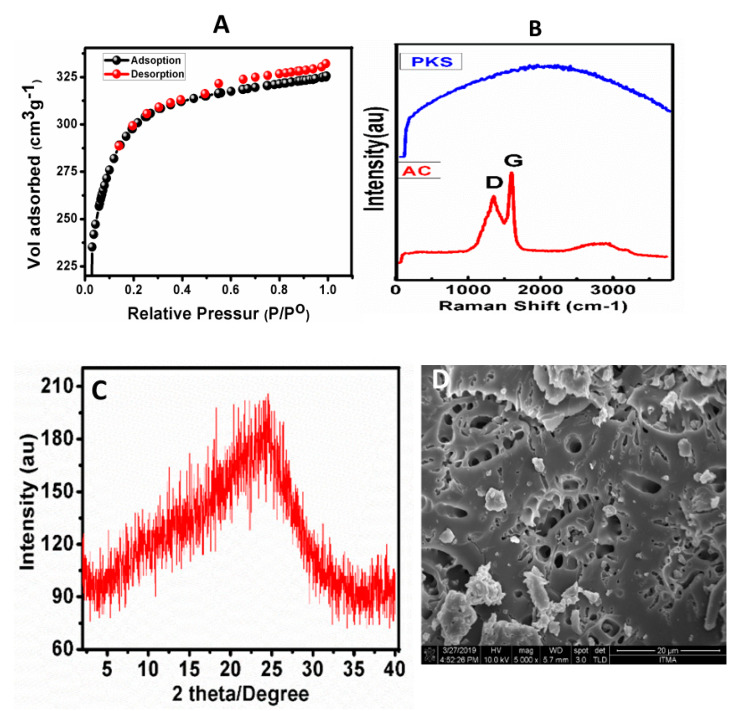

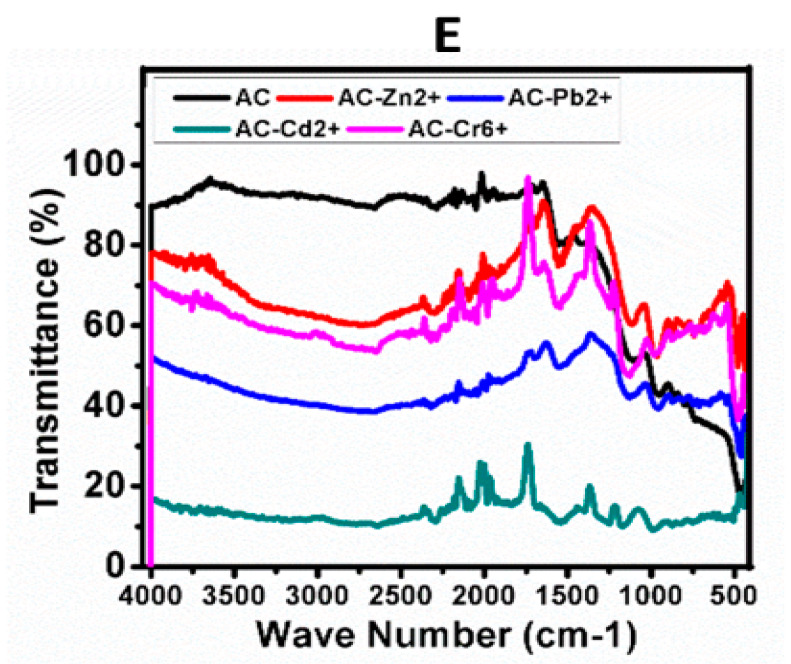
The nitrogen adsorption-desorption isotherms (**A**), Raman spectra (**B**), XRD spectra (**C**) and FESEM surface morphology of the activated carbon (**D**). The infrared spectra of the activated carbon before and after treatment with the aqueous solution of heavy metal ions is shown in (**E**).

The adsorption and desorption isotherms of the samples were obtained using the nitrogen gas method. The applied pressure and volume of the gas adsorbed and desorbed was measured. The sample was degassed for 9 h at 290 °C under a vacuum. Figure 2A shows the N_2_ adsorption-desorption isotherms of activated carbon, which follow the Type I isotherm according to the International union for pure and applied chemistry (IUPAC) classification [25]. This suggests that the synthesized activated carbon was microporous with a surface area of 1099 m^2^/g and the average pore size of 18 Å. The surface area of the PKS powder was found to be 2.8 m^2^/g, with an average pore diameter of 118 Å. 

### 3.3. Raman Spectroscopic Analysis

In this work, the Raman spectroscopy is specifically applied for the characterization of carbon-based materials because of its sensitivity to different carbon structures [17]. For example, the Raman band positions for diamond, graphite and other amorphous carbon rely upon the stress present in the carbon structure and their crystalline size [17,26]. The G band, corresponding to the presence of sp^2^ carbon in graphite-like structures, and the D band is referred to as disorders/defects in graphitic structures [18]. In this study, Raman spectroscopy was used for the characterization of activated carbon. Figure 2B shows the Raman spectra of PKS powder and activated carbon. In the Raman spectrum of PKS, no Raman band was found due to the absence of graphitic carbon; rather, a hub was observed (see Figure 2B, blue color). The Raman spectrum of activated carbon shows characteristics of the D and G bands at about 1350 cm^−1^ and 1595 cm^−1^, respectively (Figure 2B red color). The appearance of the D and G bands confirmed the successful conversion of PKS to activated carbon. The intensity ratio (I_D_/I_G_) of the D and G bands is related to defects in the graphitic structure of the activated carbon; the higher the intensity ratio, the greater the defects. The I_D_/I_G_ ratio for the synthesized activated carbon was found to be 1.30, which indicates a high degree of graphitic defects in the samples, similar to the results which have been reported previously [18]. The value of the I_D_/I_G_ ratio of about 1.27 was also related to a higher surface area. Fariz et al., conducted detailed optimization experiments and found that the highest ratio of I_D_/I_G_ of 1.305 had the highest surface area of 1169 m^2^/g [18]. Similarly, in this study, the I_D_/I_G_ ratio was found to be 1.27, having a surface area of 1099 m^2^/g.

### 3.4. X-ray Diffraction and FESEM Analysis

Figure 2C shows the XRD pattern of the prepared activated carbon. It can be observed that no specific peak was observed. A hump was observed at a 2ϴ degree of 5–35° for the activated carbon. Similar XRD results have been reported for the activated carbon prepared previously [18]. Figure 2D shows the FESEM surface morphology of the activated carbon prepared using 20% H_3_PO_4_ and carbonized at 500 °C for 2 h. The resulting material was found to show a porous structure. However, the surface morphology of the PKS showed a rough layer without any pores, as reported previously [1]. The change in the morphology from a rough layer to a porous structure suggests the successful transformation of PKS into the activated carbon. These XRD and FESEM data complement the Raman spectroscopic results, indicating the formation of the activated carbon. 

### 3.5. Infrared Spectroscopic Analysis

Infrared analysis was carried using a Fourier transformed infrared spectrometer. Figure 2E shows the infrared spectra of the activated carbon before and after treatment with heavy metal-contaminated water. The hydroxyl bands are found to be around 2500–3000 cm^−1^, with slight shifts in position after the attachment of the heavy metal ions. This OH band can be attributed to the activation of the PKS carbon with H_3_PO_4_ and moisture in the sample [16,17,18,27]. The bands at 1650–1400 cm^−1^ can be attributed to the aromatic carbon-carbon double bonds (C=C) of the activated carbon [18]. The infrared absorption bands at 1200–1100 cm^−1^ are due to CH_2_ and CH_3_ absorption bands. Furthermore, C–O and C–C stretching bands appear at 950–870 cm^−1^ and 800–735 cm^−1^, respectively [17,18,19,27]. Table 1 shows the details of the assigned infrared bands before and after treatment with heavy metal-contaminated water. There are slight shifts in the positions of the infrared bands which can be ascribed to the adsorption of heavy metal ions on the active sites of the activated carbon.

### 3.6. Batch Studies

**Figure 3:**
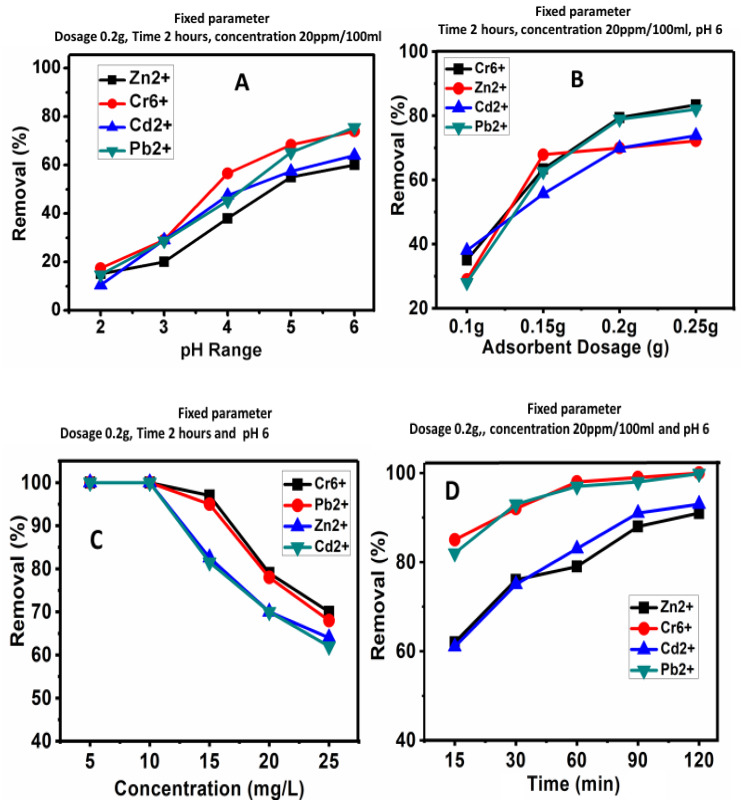
The effect of pH (**A**), adsorbent dosage (**B**), metal ions concentration (**C**) and contact time (**D**) on the adsorption process of the metal ions on the AC.

pH Effect: 

pH is the most sensitive parameter in the adsorption studies. Figure 3A shows the effect of pH on the percentage of adsorption of Cr^6+^, Pb^2+^, Zn^2+^ and Cd^2+^ metal ions on the AC. It can be observed that the adsorption of metal ions significantly increased as the pH shifted from 2 to 6. The maximum pH was set at 6 for this experiment, as higher pHs would result in the precipitation of the metal ions [6,14]. The maximum percentage adsorptions for Cr^6+^, Pb^2+^, Cd^2+^ and Zn^2+^ were found to be 78%, 80%, 63% and 60%, respectively, at pH 6. The linkage of metal ions toward the surface of the AC adsorbent is highly affected by the pH of the solution. At low pHs, metal ion adsorption was found to be lower due to H^+^ ions exchange hindrance, while at higher pHs, adsorption increased due to a greater ratio of positive metal ions [28,29]. Based on this result, for the rest of the batch experiments, a pH of 6 was used.

Effect of Adsorbent Dosage:

Figure 3B also shows the effect of the adsorbent dosage on the removal of the heavy metal ions. Different amounts of AC were used; 0.1 g, 0.15 g, 0.2 g and 0.25 g, with the initial metal ion concentration of 20 ppm in 100 mL liquid. The adsorption of metal ions was found to increase with an increase in the amount of AC. The maximum adsorption for Cr^6+^and Pb^2+^ was found to be above 80%, and for Cd^2+^ and Zn^2+^, it is above 70% at 0.25 g adsorbent/100mL. Overall, the adsorption was found to increase with the increasing of the adsorbent dosage, possibly due to the maximum number of active sites, which may be responsible for the removal of more ions at their surfaces [1,6]. The percentage adsorption of AC at 0.2 g and 0.25 g was more or less the same, therefore, for the rest of the experiments, the 0.2g adsorbent dosage was selected. 

### 3.7. Concentration Effect

The initial metal ion concentration significantly affects the adsorption of metal ions on AC. Figure 3C shows the effect of the concentration of metal ions (5 ppm, 10 ppm, 15 ppm, 20 ppm and 25 ppm) on the adsorption process. The adsorption of metal ions decreases with the increase in the metal ion concentration due to the saturation of adsorbent sites. All the metal ions showed good adsorption (97–80%) up to 15 ppm; after that, at higher concentrations; 20 ppm and 25 ppm, adsorption decreased to 80–70%. Therefore, to determine the effect of contact time, a metal ion concentration of 15 ppm was used. 

### 3.8. Time Effect

Contact time is one of the major parameters that govern the adsorption processes. Figure 3D shows the adsorption of Cr^6+^, Pb^2+^, Zn^2+^ and Cd^2+^ metal ions for the different time points; 15, 30, 60, 90 and 120 min. It was found that adsorption increases with an increase in contact time. About 99% adsorption was observed for Cr^6+^ and Pb^2+^ ions, and about 90% for Zn^2+^ and Cd^2+^ ions, at a contact time of 120 min. It can be seen that initially, there was rapid adsorption of metal ions, which can be ascribed to the free surface sites available for interactions; the process was slower over time due to reduced binding site availability [29]. 

## 4. Adsorption Kinetics 

The adsorption kinetics of metal ions on activated carbon (AC) was determined by applying different models; the pseudo-first-order, pseudo-second-order and parabolic diffusion. The kinetic equation for the pseudo-first-order in its linear form is given as follows:

ln (q_e_ − q_t_) = ln q_e_ − k_1_ t (1)

where equilibrium adsorption is represented by q_e_ and q_t_ is adsorption at equilibrium and time t, respectively and k_1_ is the rate constant, which can be determined by the slope by plotting ln (q_e_ − q_t_) versus t.

The pseudo-second-order kinetic equation can be written in its linear form as follows [30,31,32]:

(t/q_t_ − t/q_e)_ = 1/k_2_ q_e_^2^ (2)

The parabolic diffusion equation can be written as follows:

1 − (M_t_/M_o_)/t = Kt ^−0.5^ + b (3)

where M_o_ and M_t_ are adsorbed amount at time 0 and t, respectively [1,20,31,32].

Kinetic fitting for the pseudo-first-order, pseudo-second-order and parabolic diffusion models for the adsorption of metal ions by AC are shown in Figure S1 (supplementary materials). The adsorption on AC follows the pseudo-first-order for all the metal ions as the correlation coefficient, R^2^ was found to be 0.99, and the correlation coefficients for the other two models were smaller than this value. The good pseudo-first-order fitting indicated that the chemisorption process is responsible for the attachment of metal ions to the AC surface [1,26,33,34].

### 4.1. Isotherms Models of Adsorption

Adsorption interactions between metal ions and AC were determined using the Freundlich and Langmuir models. Freundlich isotherms (Equation (4)) and Langmuir isotherms (Equation (6)) can be written as follows.

Q_eq_ = K_f_ × C_eq_^(1/n)^ C_e_ (4)

The linear form:

Log Q_eq_ = logK_f_ + (1/n) × logC_e_ (5)

Q_e_ = (b Q_m_C_e_)/(1 + bC_e_) (6)

The linear form:

C_e_/Q_e_ = (C_e_/Q_m_) + 1/bQ_m_) (7)

where the equilibrium concentration of metal ions (mg/L) is represented by C_e_ and the amounts of metal ions (mg/g) adsorbed are denoted by Q_e_. The Q_m_ is the maximum amount of metal ions adsorbed (mg/g) on the AC, b is a constant (L/mg) and K_f_ (mg/g) and 1/n are the Freundlich coefficients. Figure S2 (supplementary materials) shows the isotherms data fitted with Langmuir and Freundlich isotherms models; the results are also summarized in Table 2. For all the four metal ions, the Freundlich isotherm model was found to be the best fit, as the correlation coefficient was found to be 0.99, higher than that of the Langmuir isotherm model.

### 4.2. Adsorption Capacity

**Figure 4:**
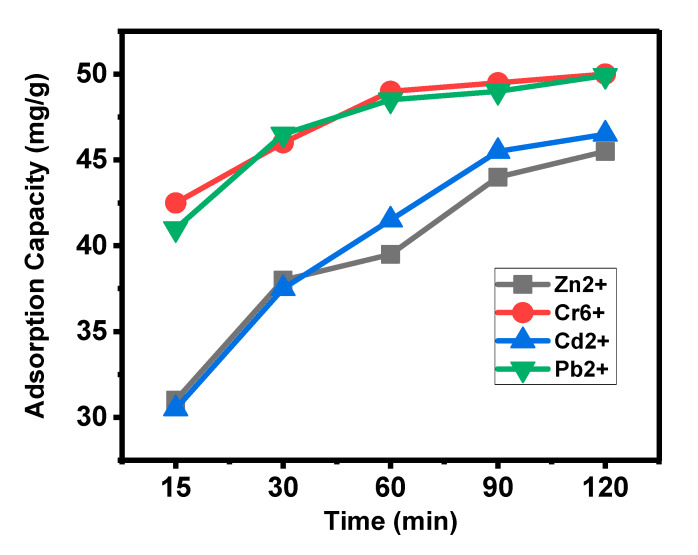
The adsorption capacity (q_e_) by the activated carbon at different time points for the metal ions Cr^6+^ Pb^2+^, Cd^2+^and Zn^2+^.

Adsorption capacity (q_e_) was analyzed by varying the contact time while keeping all the other parameters constant. Figure 4 depicts the adsorption capacity trends for time. It was found that for Cr^6+^ and Pb^2+^, they reached almost the maximum adsorption at 60 min, followed by almost a straight line due to the saturation of the adsorption sites. In contrast, Zn^2+^and Cd^2+^ took 120 min to reach the maximum adsorption, with relatively rapid adsorption trends in the first 90 min compared to the last 30 min. Adsorption capacity (Figure 4) was found to be in the order; Cr^6+^ > Pb^2+^ > Cd^2+^ > Zn^2+^. The unit of time is minutes. The adsorption capacity, q_e_ (mg/g) is given in Table 2. 

### 4.3. Comparison of PKS-Derived Activated Carbon Material with Other Carbonaceous Materials

Different carbonaceous materials have been used for the removal of heavy metals, namely single-wall carbon nanotubes (SWCNTs), functionalized-SWCNTs, multiwall carbon nanotubes (MWCNTs), functionalized-MWCNTs, graphene and graphene oxide, etc. [35,36,37,38,39,40,41,42,43,44,45,46,47,48,49]. A comprehensive review was recently published on the application of carbon nanomaterials in water treatment and environmental remediation [14]. A comparison of activated carbon with other carbon nanomaterials for the treatment of heavy-metal-contaminated water is summarized in Table 3.

## 5. Conclusions

In this study, an agricultural waste, palm kernel shell was used to prepare activated carbon. Heavy metal ions; Cr^6+^, Pb^2+^, Cd^2+^ and Zn^2+^-contaminated water were treated with the activated carbon of the highest surface area prepared from PKS by activation with 20% phosphoric acid. The parameters which influence the adsorption process, namely pH, metal ion concentration, contact time and adsorbent dosage, were optimized. The prepared activated carbon adsorbent was found to remove Cr^6+^ and Pb^2+^ ions up to about 99%, and above 80% adsorption was observed for Cd^2+^ and Zn^2+^ ions. The adsorbent can easily remove Cr^6+^ and Pb^2+^ ions a concentration of up to 20 ppm, whereas, for the removal of Cd^2+^ and Zn^2+^, a maximum ion concentration of 15 ppm may be applied for 100% removal efficiency under the optimum conditions. The first-order kinetic model was found to fit the adsorption process. An analysis of isotherms revealed that the Freundlich isotherm model fitted best compared to the Langmuir model for the adsorption process. The high surface area, porous structure and active sites on the prepared AC make it a suitable material to be used for the treatment of heavy-metal-contaminated water. This is an environmentally-friendly approach for the treatment of toxic heavy metal ion-contaminated water.

## Figures and Tables

**Table 1:** Assignment of the infrared bands of the activated carbon before and after treatment with heavy metal-contaminated water.

Assignment	AC	AC-Cr^6+^	AC-Cd^2+^	AC-Pb^2+^	AC-Zn^2+^
V (O-H)	2658	2651	2639	2600	2610
C=C (aromatic)	1687	1683	1697	1699	1728
	1554	1544	1531	1552	1556
	1418	1401	1392	1395	1393
CH_2_/CH_3_ (sym)	1205	1241	1249	1232	1235
	1104	1126	1176	1143	1119
C–O (stretching)	951	980	988	963	964
C–C (stretching)	878	878	874	874	874
	788	801	812	797	792
	735	739	743	735	735

**Table 2:** The adsorption isotherm data of Langmuir and Freundlich for Cr^6+^, Pb^2+^, Zn^2+^ and Cd^2+^ on the activated carbon.

Metal Ion	Langmuir Isotherm			Freundlich Isotherm		
	q_e_ (mg/g)	b (L/mg)	R^2^	K_f_	n_f_	R^2^
Cr ^6+^	50.76	0.010	0.9808	0.34	0.664	0.9920
Pb^2+^	50.80	0.001	0.9548	1.00	0.306	0.9993
Cd^2+^	47.01	0.101	0.4047	1.00	0.301	0.9999
Zn^2+^	45.34	0.016	0.9399	1.00	0.301	0.9999

**Table 3:** Comparison of the activated carbon with other carbon nanomaterials in the treatment of heavy-metal-contaminated water.

No.	Adsorbent	Metal Ions	Contact Time in h	Optimum pH for Adsorption	Adsorption Capacity (mg/g)/Efficiency (%)	Reference
1	AC (20% H_3_PO_4_)	Cr^6+^ Pb^2+^ Cd^2+^ Zn^2+^	2	6	99% 99% 80% 80%	Rabia et al. (This study)
2	SWCNTs	Cr^6+^	1.00	2.5	2.35 mg/g	Dehghani et al., 2015 [50]
3	MWCNTs	Cr^6+^	1.00	2.5	1.26 mg/g	Dehghani et al., 2015 [50]
4	Functionalized MWCNTs	Pb^2+^ Ni^2+^ Cu^2+^ Cd^2+^	6.00	9.0	93% 83% 78% 15%	Farghali et al., 2017 [35]
5	Functionalized MWCNTs	Cr^3+^	3.00	6.0	99.83%	Ahmad et al., 2015 [51]
6	Al_2_O_3_-MWCNTs	Pb^2+^	1.00	7.0	90%	Gupta et al. 2011 [52]
7	Porous graphene	As ^3+^	1.00	7.0	90%	Tabish et al., 2018 [53]
8	rGO-Fe_3_O_4_	Pb^2+^	0.16	6.0	373.14 mg/g	Guo et al., 2018. [54]
9	(reduced GO-Sulfophenylazo (rGOS)	Pb^2+^ Cu^2+^ Ni^2+^ Cd^2+^ Cr^3+^	0.16	5.0	689 mg/g 59 mg/g 66 mg/g 267 mg/g 191 mg/g	Zhang et al., 2018 [40]

## Supplementary Materials

The following are available online at https://www.mdpi.com/1996-1944/13/11/2627/s1, Figure S1. Kinetic graphs of Pseudo first order, Pseudo second order and parabolic diffusion for the adsorption Cr^6+^, Pb^2+^, Zn^2+^ and Cd^2+^ metal ions on the AC. Figure S2. Freundlich and Langmuir Isotherms models-adsorption metal ion Cr^6+^, Pb^2+^, Cd^2+^ and Zn^2+^ on the activated carbon.
