# Supplementary material for: Ecofriendly Approach for Treatment of Heavy-Metal-Contaminated Water Using Activated Carbon of Kernel Shell of Oil Palm
Source: Materials (Basel). 2020 Jun 9;13(11):2627. doi: 10.3390/ma13112627 (PMC7321607; doi:10.3390/ma13112627)
Supplement: Supplementary file 1 [file materials-13-02627-s001.pdf]

materials

Supplementary Materials: Ecofriendly Approach for Treatment of Heavy-Metal-Contaminated Water Using Activated Carbon of Kernel Shell of Oil Palm

Rabia Baby and Mohd Zobir Hussein *

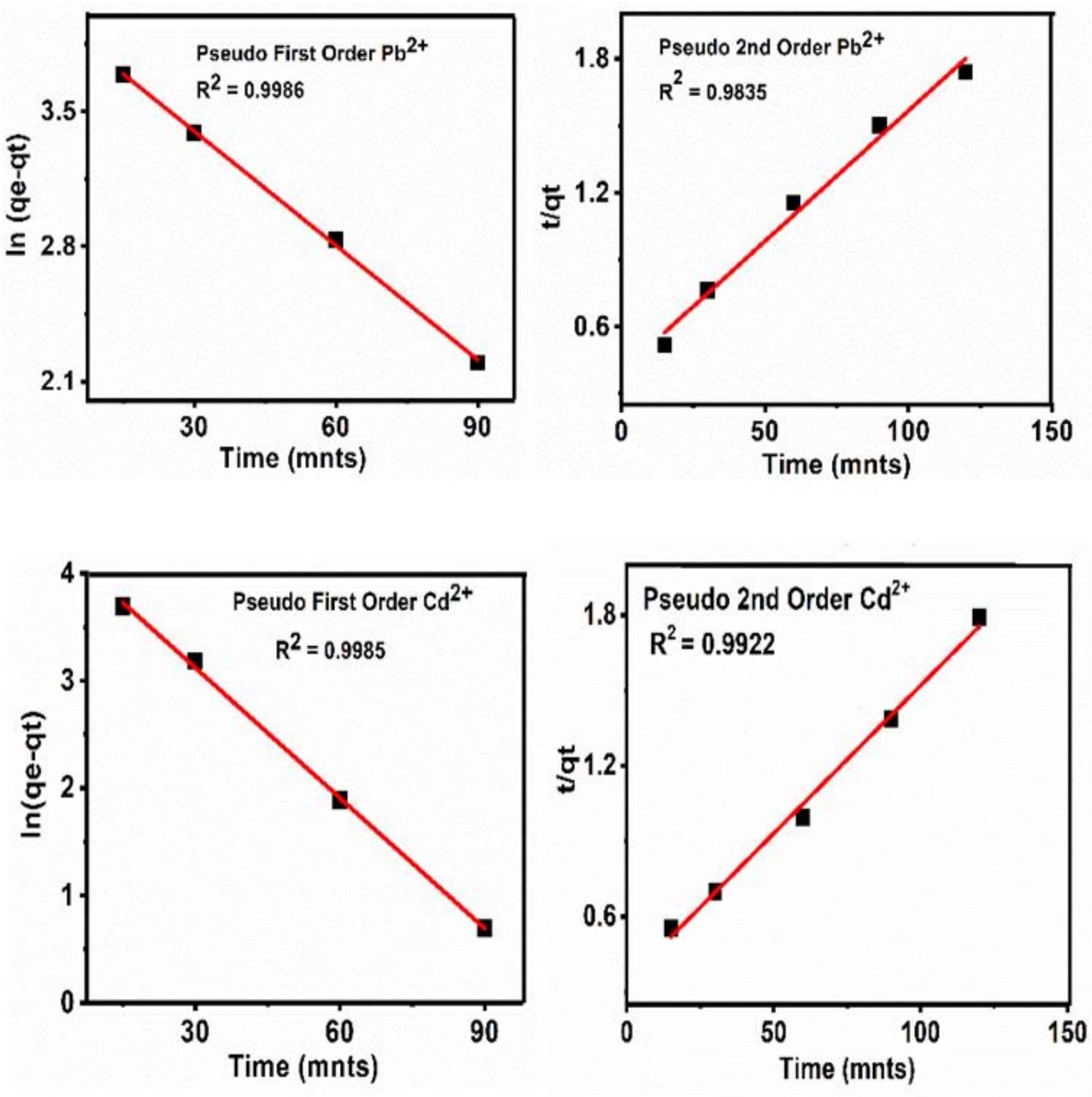

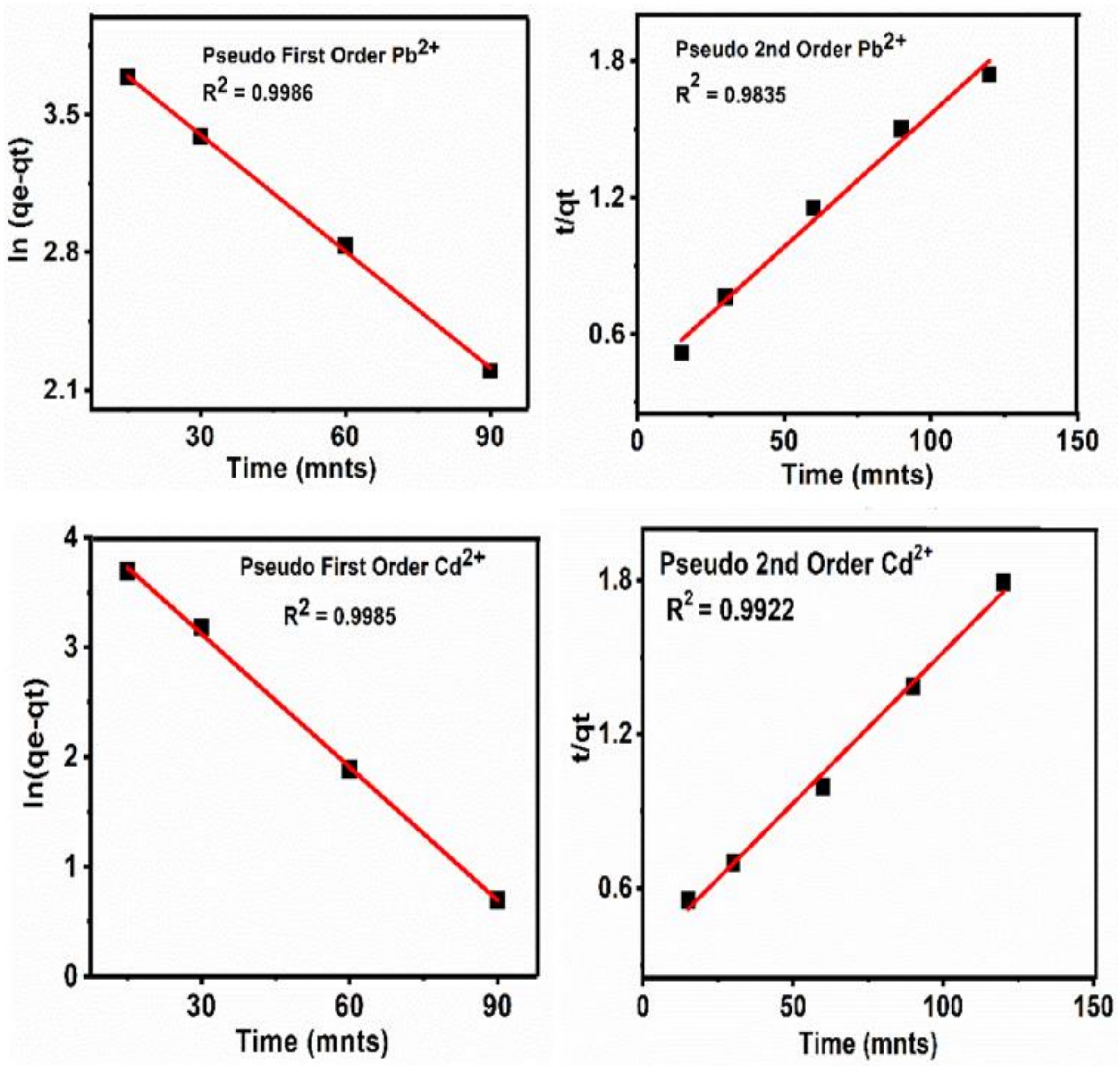
Pseudo First Order Pb2+
R2 = 0.9986
ln (qe-qt)
3.5
2.8
2.1
30
60
90
Time (mnts)
Pseudo 2nd Order Pb2+
R2 = 0.9835
t/qt
1.8
1.2
0.6
0
50
100
150
Time (mnts)
Pseudo First Order Cd2+
R2 = 0.9985
ln(qe-qt)
4
3
2
1
0
30
60
90
Time (mnts)
Pseudo 2nd Order Cd2+
R2 = 0.9922
t/qt
1.8
1.2
0.6
0
50
100
150
Time (mnts)

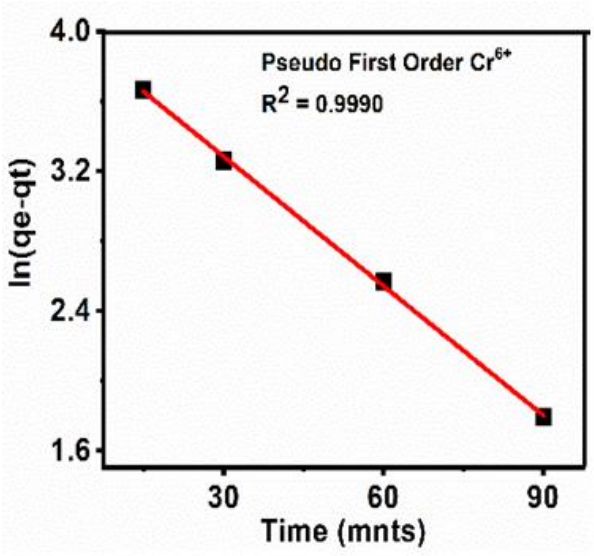
Pseudo First Order Cr^{6+}
$R^2 = 0.9990$
ln(qe-qt)
4.0
3.2
2.4
1.6
30
60
90
Time (mnts)

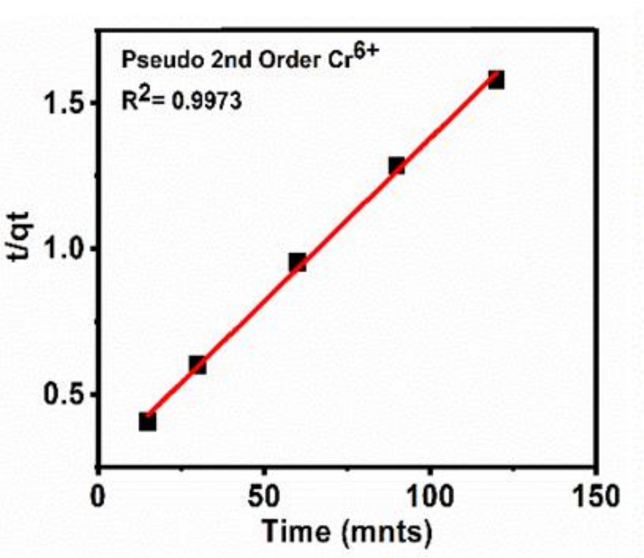
Pseudo 2nd Order Cr^{6+}
$R^2 = 0.9973$
t/qt
1.5
1.0
0.5
0
50
100
150
Time (mnts)

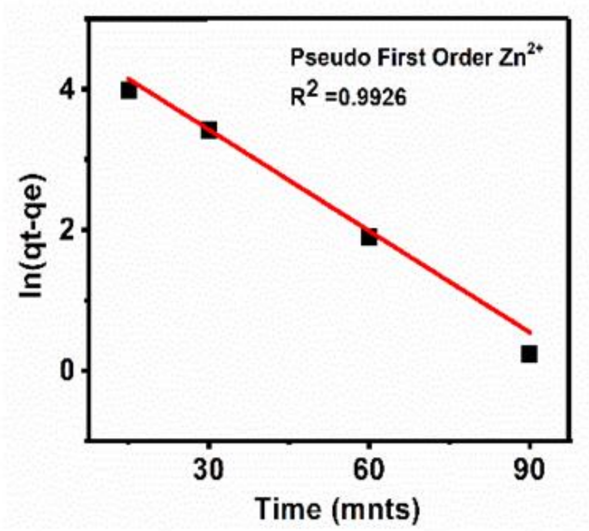
Pseudo First Order Zn^{2+}
$R^2 = 0.9926$
ln(qt-qe)
4
2
0
30
60
90
Time (mnts)

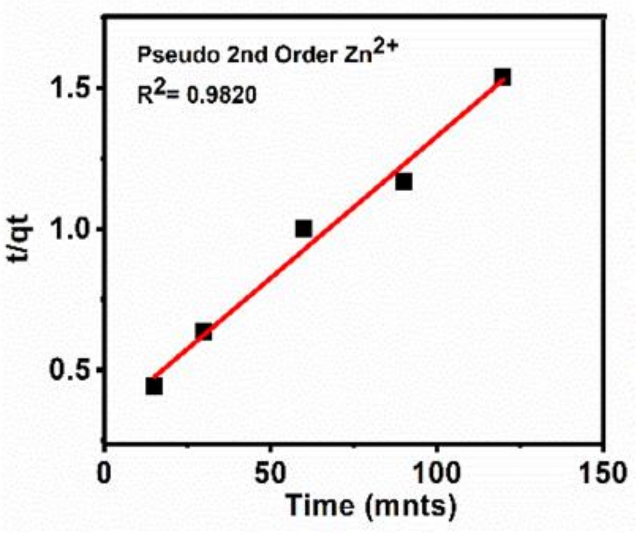
Pseudo 2nd Order Zn^{2+}
$R^2 = 0.9820$
t/qt
1.5
1.0
0.5
0
50
100
150
Time (mnts)

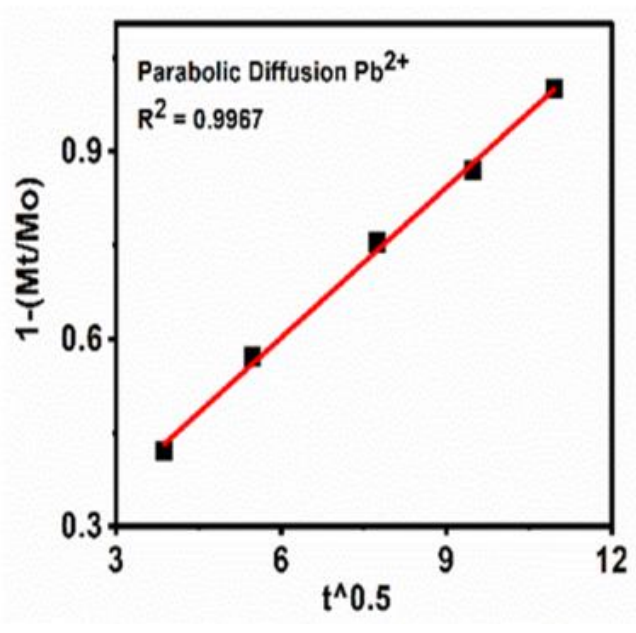
Parabolic Diffusion Pb^{2+}
$R^2 = 0.9967$
1-(Mt/Mo)
0.9
0.6
0.3
3
6
9
12
t^0.5

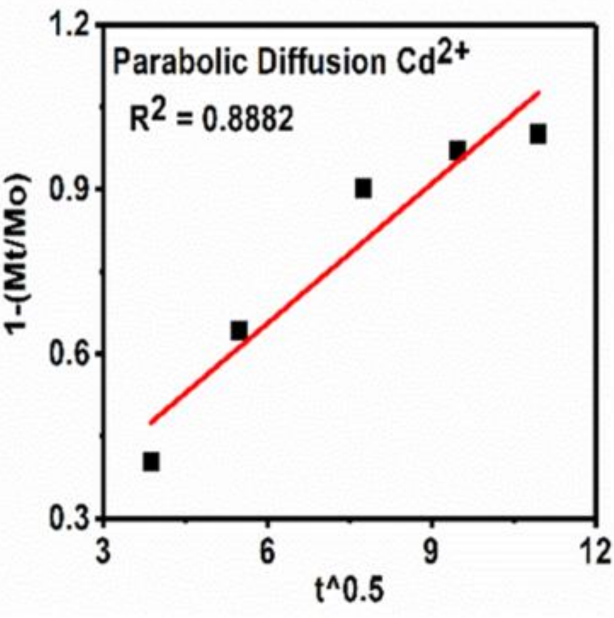
Parabolic Diffusion Cd^{2+}
$R^2 = 0.8882$
1-(Mt/Mo)
1.2
0.9
0.6
0.3
3
6
9
12
t^0.5

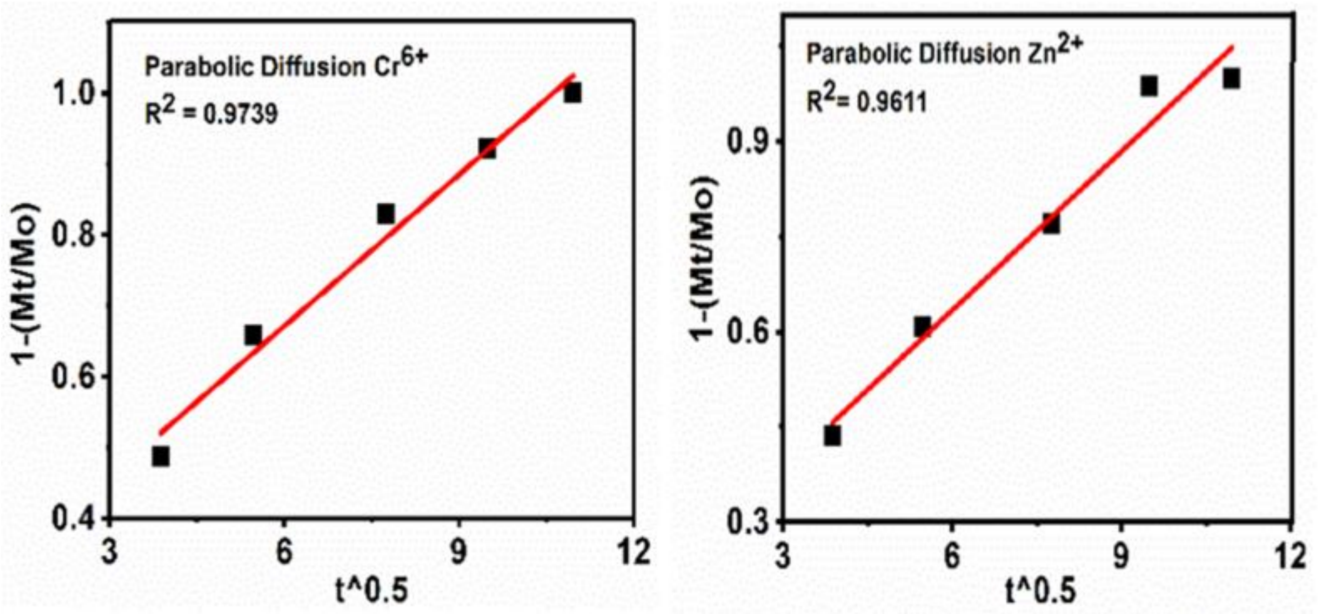

Figure S1. Kinetic graphs of Pseudo first order, Pseudo second order and parabolic diffusion for the adsorption Cr+6, Pb+2, Zn+2 and Cd+2 metal ions on the AC.

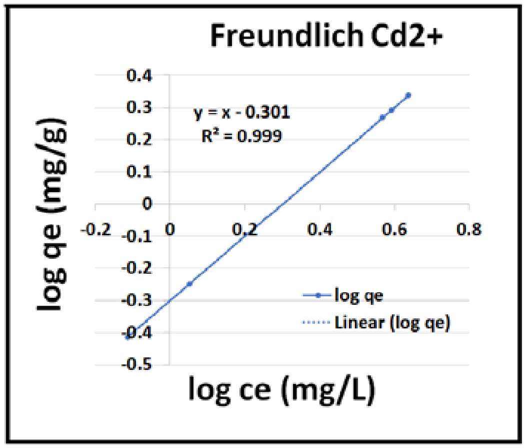
Freundlich Cd2+
y = x - 0.301
R² = 0.999
log qe (mg/g)
log ce (mg/L)
log qe
Linear (log qe)

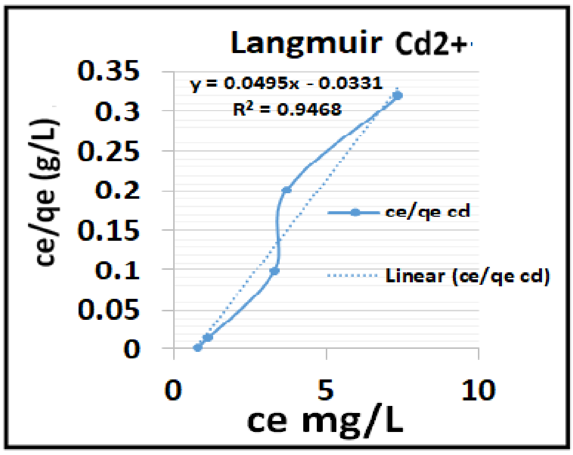
Langmuir Cd2+
y = 0.0495x - 0.0331
R² = 0.9468
ce/qe (g/L)
ce mg/L
ce/qe cd
Linear (ce/qe cd)

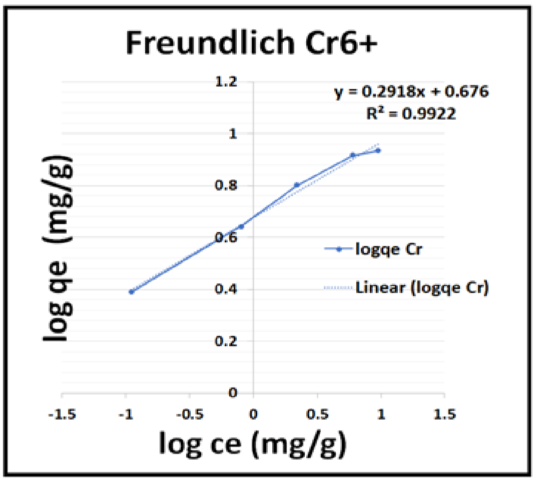
Freundlich Cr6+
y = 0.2918x + 0.676
R² = 0.9922
log qe (mg/g)
log ce (mg/g)
logqe Cr
Linear (logqe Cr)

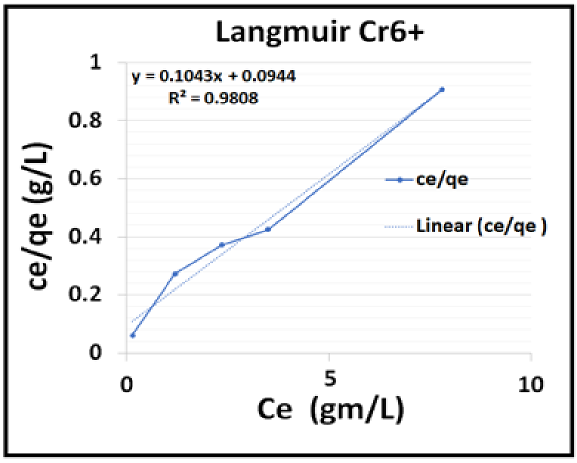
Langmuir Cr6+
y = 0.1043x + 0.0944
R² = 0.9808
ce/qe (g/L)
Ce (gm/L)
ce/qe
Linear (ce/qe)

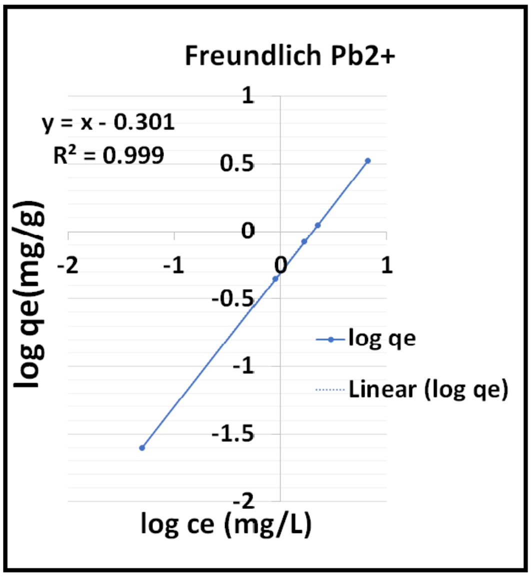
Freundlich Pb2+
y = x - 0.301
R² = 0.999
log qe(mg/g)
log ce (mg/L)
log qe
Linear (log qe)

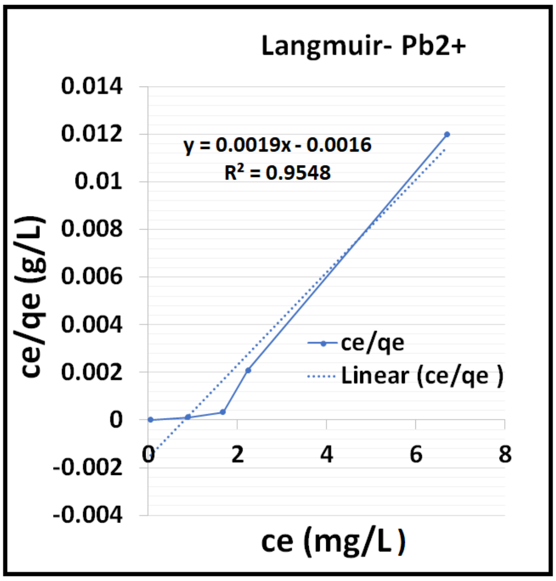
Langmuir- Pb2+
y = 0.0019x - 0.0016
R² = 0.9548
ce/qe (g/L)
ce (mg/L)
ce/qe
Linear (ce/qe)

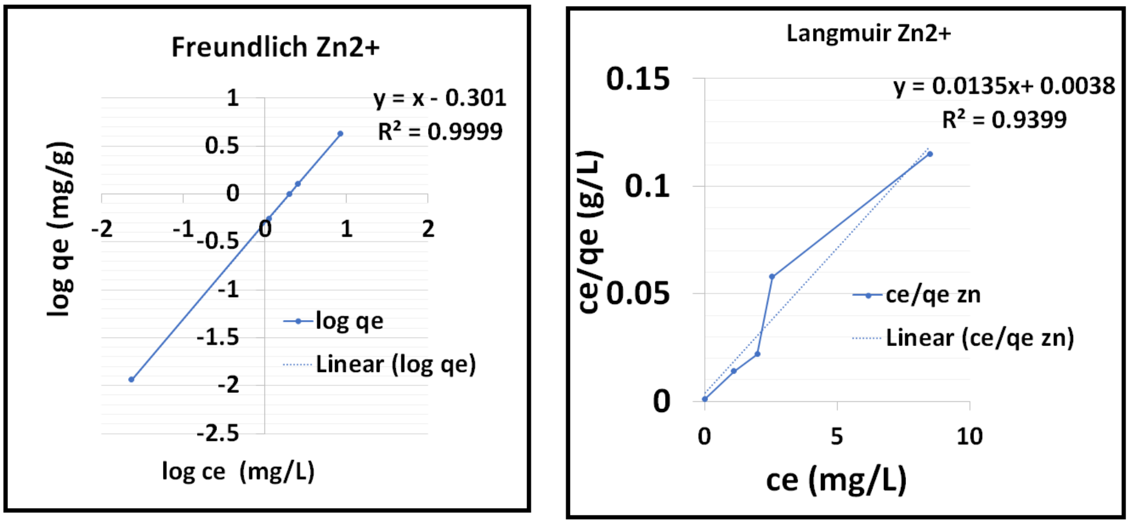

Figure S2. Freundlich and Langmuir Isotherms models-adsorption metal ion Cr^{6+}, Pb^{2+}, Cd^{2+} and Zn^{2+} on the activated carbon.

© 2020 by the authors. Licensee MDPI, Basel, Switzerland. This article is an open access article distributed under the terms and conditions of the Creative Commons Attribution (CC BY) license (http://creativecommons.org/licenses/by/4.0/).
